# Insight for Immunotherapy of HCMV Infection

**DOI:** 10.7150/ijbs.58127

**Published:** 2021-07-13

**Authors:** Xinmiao Long, Yi Qiu, Zuping Zhang, Minghua Wu

**Affiliations:** 1 Hunan Cancer Hospital and the Affiliated Cancer Hospital of Xiangya School of Medicine, Central South University, Changsha 410013, Hunan, China; 2 The Key Laboratory of Carcinogenesis of the Chinese Ministry of Health, The Key Laboratory of Carcinogenesis and Cancer Invasion of the Chinese Ministry of Education, Cancer Research Institute, Central South University, Changsha, 410008 , Hunan, China; 3 Department of Pathogeny Biology, School of Basic Medical Science, Central South University, Changsha, 410078, Hunan, China

**Keywords:** human cytomegalovirus, immunotherapy, immune response, immune escape mechanism, infection

## Abstract

Human cytomegalovirus (HCMV), a ubiquitous in humans, has a high prevalence rate. Young people are susceptible to HCMV infection in developing countries, while older individuals are more susceptible in developed countries. Most patients have no obvious symptoms from the primary infection. Studies have indicated that the virus has gradually adapted to the host immune system. Therefore, the control of HCMV infection requires strong immune modulation. With the recent advances in immunotherapy, its application to HCMV infections is receiving increasing attention. Here, we discuss the immune response to HCMV infection, the immune escape mechanism, and the different roles that HCMV plays in various types of immunotherapy, including vaccines, adoptive cell therapy, checkpoint blockade therapy, and targeted antibodies.

## Introduction

Human cytomegalovirus (HCMV), also known as human herpesvirus 5 (HHV-5), is a linear double-stranded DNA β-herpesvirus that belongs to the family of herpesviruses 1. HCMV infection results in the enlargement of infected tissue cells and the formation of large nuclear inclusion bodies. HCMV was first isolated in 1957 from an infant who was suspected to have congenital toxoplasmosis 2.

A recent systematic review and meta-analysis estimated a global HCMV seroprevalence of 83% in the general population, 86% in women of reproductive age, and 86% in donors of organs or blood 3. HCMV can be transmitted through mother-to-child transmission; horizontal transmission; hospital-acquired respiratory, neurological, blood, and digestive system diseases; and other multi-system diseases, resulting in fetal malformation, birth defects 4, or poor prognosis of transplant patients with low immunity 5-8. Following the establishment of infection, HCMV is usually latent (with only a few virus particles being released outside the cell), and the infected persons remain asymptomatic and become lifelong infectious carriers 9. However, it is a huge health threat for immunocompromised patients, such as newborns 10, 11, AIDS patients 12, and transplant recipients 13. It often causes infectious mononucleosis-like syndrome, retinitis, pneumonitis, gastrointestinal diseases, mental retardation, and vascular disorders 14. Interestingly, recent studies have reported the presence of active HCMV infection in gliomas 15 and breast cancer 16. HCMV infection induces the activation of mitotic signals transmitted by the products of proto-oncogenes such as c-fos c-jun and c-myc, which may be involved the tumorigenicity of HCMV 17.

Currently used drugs, such as Ganciclovir (GCV), Cidofovir (CDV) and Foscavir (FOS), have problems such as poor antiviral effect, large side effects and drug resistance, which cannot meet the clinical needs. Therefore, it is urgent to develop new prevention and treatment methods 18. Antiviral drugs used in the treatment of patients with HCMV infection have low oral bioavailability and dose-related toxicities, such as bone marrow suppression, hepatotoxicity, and nephrotoxicity 19, 20. In addition, vaccines against HCMV are being developed, but no licensed vaccine is available thus far 21. Fortunately, a number of studies have shown that immunotherapy is a promising strategy to overcome the side effects of antiviral treatment and for the development of prevention measures.

## The structural characteristics of HCMV

The virion of HCMV is composed of double-stranded DNA (dsDNA), which is enclosed in a capsid comprising four integral protein (pUL46, pUL80.5, pUL85, pUL104) that are organized into 162 capsomeres (150 hexamers plus 12 pentamers) and 320 triplexes located between the capsomeres 22. The capsid, with a diameter of 100 nm, is surrounded by a poorly defined area and enclosed by a lipid bilayer envelope containing viral glycoproteins providing a final diameter of approximately 180 nm for the whole virion 23. The HCMV genome is the largest known human herpes virus genome. Recent analysis has suggested that the DNA genome of HCMV consists of 235,000 base pairs, encoding at least 700 open reading frames 24. The structure of the HCMV genome is similar to that of other herpes virus members. It is composed of the unique sequence of long component (UL) and the unique sequence of short component (US) and has reverse repeating sequences, specifically terminal repeat of long component (TRL), inverted repeat of long component (IRL), inverted repeat of short component (IRS), and terminal repeat of short component (TRS) The order of expression was immediate-early (IE), early (E), and late (L) genes. IE genes are expressed within hours after infection and initiate the transcription of viral genes essential for genome replication. E genes encode proteins, such as DNA polymerase and the terminase complex, which contribute to viral DNA replication and packaging and regulate host cell functions to facilitate virus replication. L genes encode structural proteins of the outer tegument layer and the envelope required for the assembly of the infectious virion 25. HCMV genome encodes approximately 54 membrane proteins including at least 25 membrane glycoproteins found in the virion envelope 26-28. A well-defined list of HCMV membrane proteins necessary for virus entry into the cells is not available because most HCMV mutants have been classified as essential or nonessential for replication, and have not been tested for defects in virus entry 29, 30. Glycoprotein B (gB), glycoprotein H (gH), glycoprotein L (gL), glycoprotein M (gM), and glycoprotein N (gN) are the main HCMV-encoded glycoproteins. gB is a viral fusogene that is essential for entry into all cell types, 31. Furthermore, gB (UL55) is highly expressed and an immune-dominant target following natural infection, making it an attractive target for vaccination 32. The gH/gL assemble into UL128, UL130, and UL131 proteins producing gH/gL/UL128-131, which are found in the virion envelope and mediate infection of epithelial and endothelial cells and monocyte-macrophages 33-35. Among HCMV proteins, the tegument phosphoprotein pp65 (pUL83) is a common clinically detected antigen and plays a major role in immunomodulation and immune evasion.

## The life cycle of HCMV

The life cycle of HCMV starts when it binds to receptors on the surface of host cells and enters into the cytoplasm. Current data support two models of HCMV entering the cytoplasm through receptor-mediated endocytosis and membrane fusion 36. One model involves the interaction of the trimeric gH/gL/gO complex (TC) with platelet-derived growth factor receptor alpha (PDGFRα), activating gB to fuse the virus envelope with the membrane of fibroblasts 37. The other model involves the interaction of the pentameric gH/gL/UL128-131 complex (PC) with neuropilin-2 (Nrp2), which promotes endocytosis of virus particles in epithelial cells/endothelial cells, followed by gB activation by gH/gL/gO (or gH/gL) and entry into the cytoplasm through endosomes 38. The network of microtubules (MTs) are closely related to the entry of HCMV into the nucleus. When the MTs are depolymerized by nocodazole, fluorescently labelled pp150 is blocked in the cytoplasm. Furthermore, in the absence of the MT network, the capsids that have entered the cytoplasm do not move in close proximity to the nucleus, suppressing IE gene expression 39, 40. After the entry of viral DNA into the cell nucleus, cellular RNA polymerases I and II (Pol I and II) are employed to transcribe viral genes by binding to the major immediate early promoter (MIEP) 41. Previous studies have demonstrated that inhibition of Rho-associated coiled-coil kinase (ROCK) protein 1 results in increased levels of tegument protein UL32 and viral DNA in the cytoplasm, suggesting that ROCK activity hinders efficient egress of HCMV particles out of the nucleus 42. Such impairments have been previously linked to the failure to control HCMV infection 43. In addition, The transcriptome of HCMV-infected cells showed features of a pro-oncogenic cellular environment with upregulated expression of multiple oncogenes, enhanced activation of pro-survival genes, cell proliferation, and upregulated markers of stem cells and epithelial mesenchymal transition (EMT) 44.

There are two types of cell infection status: latent infection and lysogenic infection. For a latent infection, latency-associated transcript (LAT) HCMV genes are transcribed and accumulated in host cells 45. A number of HCMV LATs have been identified, including UL138 46, latent undefined nuclear antigen (LUNA) 47, UL81-82 48, US28 49, 50, and pUL11 51. Experimentally, latently infected CD34+ and CD14+ cells have been shown to secrete chemokines, which can recruit T cells, as well as IL-10 and TGF-β, both of which can modulate the activity of T cells that have migrated to the environment surrounding the latent infection 51, 52. In this case, viruses can indefinitely persist in host cells and have a latent infection pathway. Primary infection may be accompanied by a limited illness, and long-term latency is often asymptomatic 53. Reactivation of HCMV in IL-6-stimulated dendritic cells (DC) has been reported to be dependent on ERK-MAPK pathway 54.

## The immune response of HCMV infection

The reason why we have not found an effective way to combat HCMV is probably that the immune responses to HCMV infections are still poorly understood. Changes in the level of the immune response directly affect the severity of HCMV infection. However, how the immune system responds to HCMV infection is not yet clear.

It is well known that cell-mediated immunity and antibody-mediated immunity are necessary to suppress HCMV infection 55-57.Firstly, HCMV can infect a broad range of cells, including epithelial cells of glandular and mucous tissues, smooth muscle cells, fibroblasts, macrophages 58, hepatocytes, dendritic cells 59, and vascular endothelial cells 60, all of which provide a platform for the efficient proliferation of HCMV 36. HCMV infection of precursor monocytes leads to the generation of toll-like receptors (TLRs) responsive inflammatory macrophages resistant to down-regulatory stromal TGF-β, allowing the macrophages to react to invading pathogens and immunostimulatory products with inflammation mediated by the Smad7 cytokine response 61.Within the innate immune system, natural killer (NK) cells act as the first line of defense and play an important role in limiting early cytomegalovirus (CMV) infection 62. Monocytes play a pivotal role in viral dissemination in organ tissues during primary infection and subsequent reactivation from latency 63. One hallmark of CMV infection is the maintenance of large populations of CMV-specific memory CD8+ T cells, a phenomenon termed memory inflation, and emerging data suggest that memory inflation is associated with impaired immunity in the elderly 55. HCMV can activate the expression of B cell-activating factor (BAFF-R), thereby promoting polyclonally activated B lymphocytes 64. HCMV pp65 may be an autoimmune or lupus-prone B cell epitope and may catalyze further epitope spreading to induce anti-dsDNA antibody production in lupus-susceptible individuals 65. pp65 inhibits interleukin-1β (IL-1β) in an NF-κB-dependent manner, induces IL-1β in a caspase-8 dependent manner 66, and together with IE1 antigen efficiently induces and expands virus-specific T cells 67. There are still several proteins that play important roles in HCMV infection and immunity, which are worth further exploration and research.

Currently, CD4+ or CD8+ T cells and NK cells are considered to play significant roles in the host defense against HCMV, and they are the key participants in the cellular immune response. CD4+ or CD8+ T cells restrain viral replication and prevent disease, but do not eliminate the latently infected host cell. How do latently infected cells pose a health risk from a potential reactivation? HCMV may be reactivated by immunosuppression, inflammation, differentiation, or critical diseases 9, to begin generating a large number of viral progenies to cause symptoms and diseases, described as the lytic life cycle. When HCMV are reactivated, the transcription of viral genes switches from LAT genes to lytic genes to enhance viral DNA replication and virion production 45. However, it is not clear how HCMV senses changes in the surrounding immune microenvironment and proliferates. Maintaining the transcription of the LAT gene, maintaining the latent state, inhibiting lysogenic infection, and thus inhibiting the progression of infection, is a good balance for stabilizing the patient.

## The immune escape mechanism of HCMV

**Figure 1:**
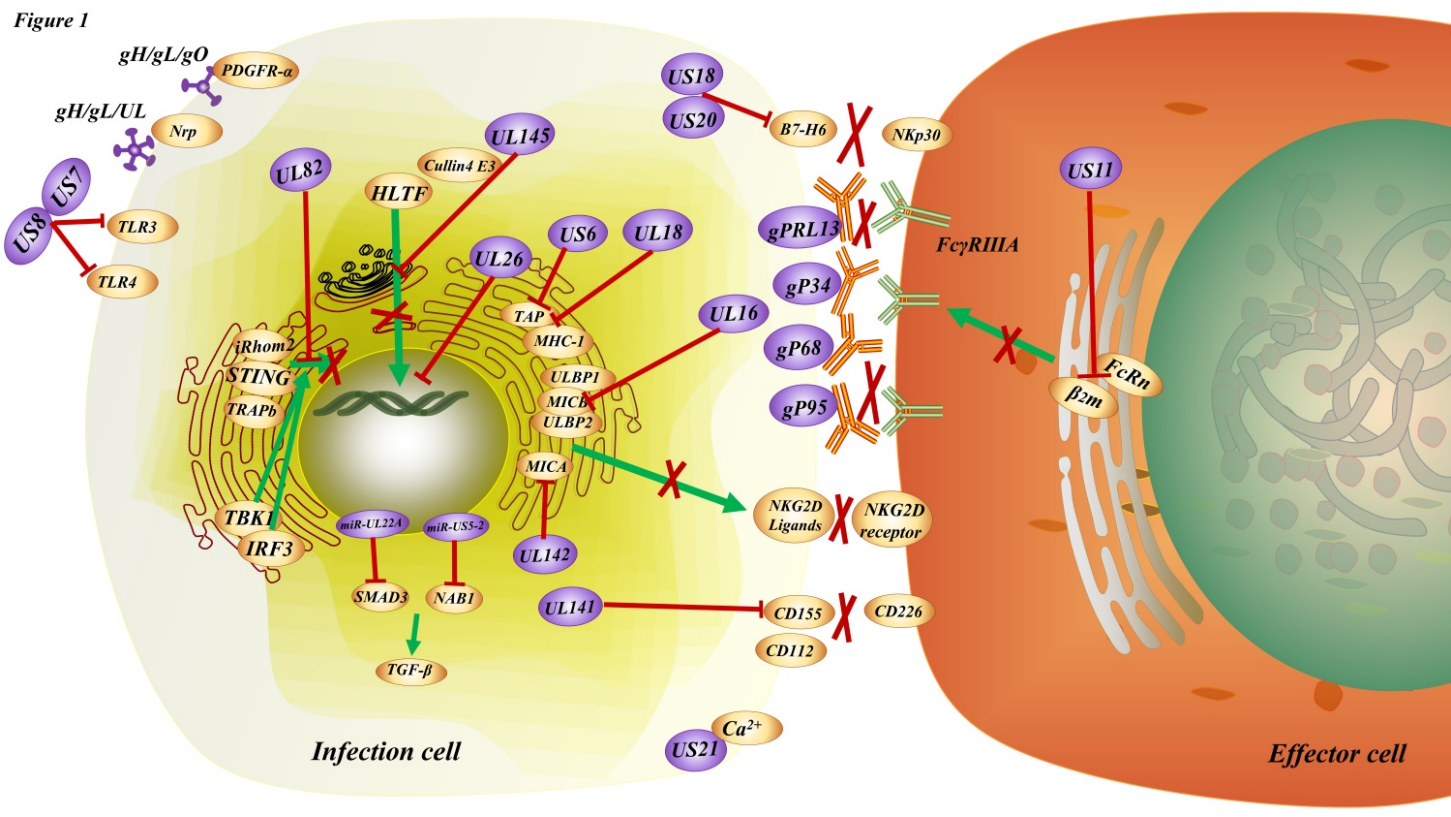
** The immune modulation induced by HCMV.** HCMV entry into the infected cells through the interaction of the trimeric gH/gL/gO complex (TC) with PDGFRα or the binding of the pentameric gH/gL/UL128-131 complex (PC) with Nrp2, etc. There are multiple strategies for the HCMV to achieve immune escape. For instance, US7 and US8 bind both TLR3 and TLR4, facilitating receptor destabilization by distinct mechanisms; US11 inhibits the assembly of FcRn with β2m resulting in the retention of FcRn in the endoplasmic reticulum, consequently blocking FcRn trafficking to the endosome; UL16, UL142 bind to ligands for NKG2D, the natural killer cell-activating receptor; UL18, US6 interfere with the physical association between MHC class I molecules and TAP; US18, US20 downregulation of B7-H6 leads to evasion from NKp30-mediated killing; US21 protein is a viral-encoded ion channel that regulates intracellular Ca^2+^ homeostasis and protects cells against apoptosis; UL26 downregulates the expression of antiviral genes; UL82 inhibits STING-mediated signaling; UL141 promotes efficient downregulation of the natural killer cell activating ligand CD112; UL145 hijacks Cullin4 to invoke HLTF; miR-UL22A and miR-US5-2 suppress the secretion of TGF-β; gp68, gP34, gpRL13 and gP95 bind to the Fc segment of IgG on the membrane, resulting in the inability of FcγRⅢA receptor on effector cells to bind to the antibody on the target cells and hinder the communication or cross talk between cellular immunity and humoral immunity.

HCMV can evade an immune response in three ways: evasion from immune recognition receptors, inhibition of immune cell activation, or suppression of effector function (Figure 1).

In terms of evading immune recognition receptors, TLRs comprise a significant signaling pathway required for antiviral defense. The HCMV-encoded glycoproteins US7 and US8 target the TLR3 and TLR4 signaling pathways by promoting the degradation of these TLRs; US7 targets TLR3 and TLR4 by a ubiquitin/proteasome system; and US8 promotes TLR3 and TLR4 destabilization. This results in the overall downregulation of TLR-mediated innate antiviral response 68. Recognition of HCMV DNA by the cytosolic sensor cyclic GMP/AMP synthase (cGAS) initiates stimulator of interferon genes (STING)-dependent innate antiviral responses. However, the HCMV tegument protein UL82 (also known as pp71) has been identified as a negative regulator of STING-dependent antiviral responses. UL82 inhibits the translocation of STING from the endoplasmic reticulum to perinuclear microsomes by disrupting the STING-iRhom2-TRAPb translocation complex. UL82 also impairs the recruitment of TANK-binding kinase 1 (TBK1) and interferon regulatory factory (IRF3) to the STING complex and impairs STING-mediated signaling 69.

In addition, in order to inhibit immune cell activation, previous studies have shown that there are many HCMV proteins that can inhibit immune cell activation, such as UL16 70, UL18 71, US6 72, US18, US20 73, US21 74, UL26 75, UL140, UL141 76-78, UL142 79, US18, US20 80 even some miRNAs 81, 82. Recent studies have shown that HCMV infection increases the expression of immune checkpoint genes encoding PD-L1, PD-L2, PD-1, CD80, CD86, Tim-3, LAG3, as well as other T-cell markers such as CD4 and CD8A in tumors along the gastrointestinal tract, including the esophagus, stomach, and intestine 83. By examining lymphocytes isolated from subjects at the time of viremia, it was found that T cells from individuals with short telomeres proliferated less, produced fewer cytokines, and had less induction of the transcription factor T-box expressed in T cells(T-bet) when stimulated with viral peptides 84. Helicase-like transcription factor (HLTF), a DNA helicase important for DNA repair, potently inhibits early viral gene expression but is rapidly degraded during infection. The HCMV protein UL145 facilitates HLTF degradation by recruiting the Cullin4 E3 ligase complex and additionally targets tumor protein p53-binding protein 1 (TP53BP1) to invoke this immune evasion strategy in the cytoplasm.

HCMV mainly breaks the link, or the interaction or the communication or the cross talk between cellular immunity and humoral immunity by inhibiting the engagement of HCMV-specific antibody Fc fragments to FcR, and then suppressing effector function. Antibody-dependent cellular phagocytosis (ADCP) occurs upon engagement of virus-specific antibody Fc fragments to FcR, resulting in the cytotoxic killing of infected cells and whole virion degradation. HCMV also encode their own viral FcRs, which recognize the Fc regions of host immunoglobulins. Mimicking host FcRs, vFcRs enable herpesviruses to reduce and evade antiviral immune responses 85. In addition, gp68 86, gp34 87, gpRL13, and gp95 88, 89, were found to be expressed on the membrane surface of HCMV infected cells, and bind to the Fc segment of IgG on the membrane 85, resulting in the inability of FcγRIIIA receptor on NK cells to bind to the antibody on the target cells and hinder the bridge between cellular immunity and humoral immunity 90, 91. One study proposed that the HCMV glycoprotein US11 inhibits neonatal Fc receptor functions (FcRn), causing its degradation in the endoplasmic reticulum, which may dampen mucosal and maternal immunity and reduce IgG half-life in blood and tissues, ultimately helping HCMV to escape antibody-mediated immunity 92.

In fact, there are multiple strategies for the “tricky” HCMV to achieve immune escape. At the same time, the elucidation of these immunosuppressive strategies of HCMV also provides ideas for controlling the progression of infection.

## Current status of immunotherapy for HCMV

**Figure 2:**
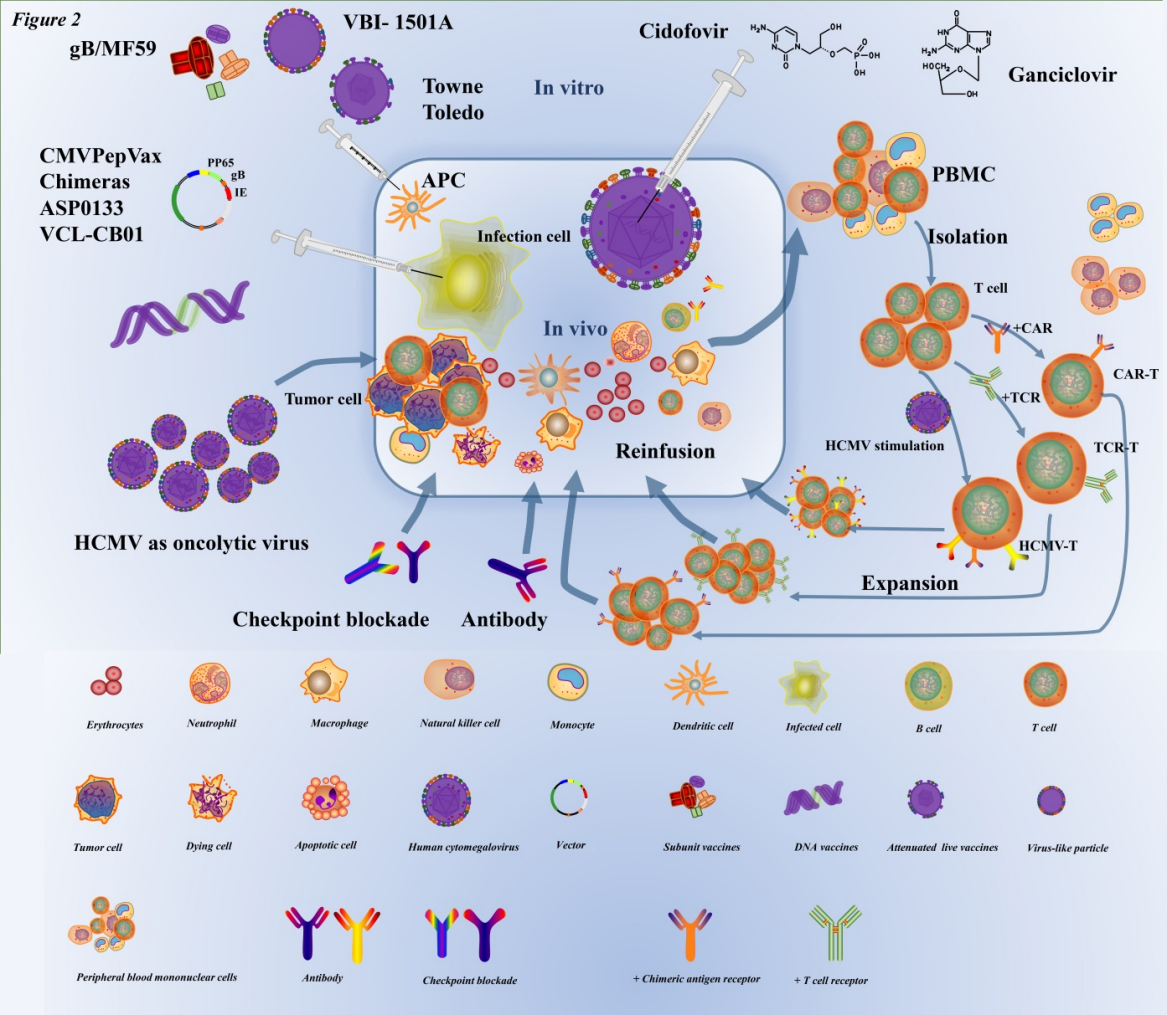
** The current prevention and therapy strategies for HCMV infection.** With respect to vaccines to prevent HCMV infection, inactivated virus (e.g., Towne, Toledo) weakened by a series of in vitro subcultures and subunit vaccines (e.g., gB/MF59), DNA vaccines (e.g., CMVPepVax, e.g., CMVPepVax, Chimeras, ASP0133, VCL-CB01), have been developed. When these vaccines are injected in the body, they can activate lymphocytes to kill the infected cell antigen-presenting cells (e.g., dendritic cells, macrophages, monocytes). A classic common antiviral inhibitor (cidofovir, ganciclovir, etc.) is a nucleotide analog that mainly impedes the DNA of HCMV synthesis. The target of checkpoint blockade therapy and targeted antibodies in HCMV infection is still under research. HCMV, as an oncolytic virus, usually infects tumor cells and creates an inflammatory microenvironment by causing infected cells to express molecules that target antigenic determinants and recruit immune cells (such as T lymphocytes, macrophages, monocytes, etc.) to cause infected cell apoptosis. ATC involves extraction of PBMC from the body, isolation of target cells (such as T lymphocytes and NK cells) in vitro genetic modification to immunotherapy cells, including HCMV-specific T cells, TCR-T cells, CAR-T cells, and then multiplication, and finally reinfusion into the body.

Immunotherapy is the treatment of diseases by artificially enhancing or suppressing the immune function of the body. Immunotherapy has been widely used in the treatment of a large number of cancers, and has achieved good results in different cancers such as the brain 93, 94, breast 95, lung 96, and ovarian cancer 97. At the same time, it must be admitted that there are still many areas for improvement in immunotherapy. HCMV, which is latent in cells, has been co-evolving with humans over the millenary, and the efficacy of antiviral drug therapy seems suboptimal. With the advancement of immunotherapy, an increasing number of researchers have applied immunotherapy for HCMV infection (Figure 2).

## Vaccines: prophylactic and therapeutic

Several types of HCMV vaccine candidates have been developed or are currently under development, including first-generation vaccines (live-attenuated Towne), second-generation vaccines (gB protein-based, peptide vaccines, virus-like particles), and third-generation vaccines (nucleic acid vaccines) 98.

The first-generation vaccine, Towne, attenuated by serial in vitro passaging, seems to have lost its ability for persistent immune induction, as indicated by gradually declining T lymphocyte responses 99. The Towne vaccine does not cause local or systemic reactions and has an faultless safety record 100, 101. However, seronegative kidney transplant recipients who received the Towne vaccine were partially protected against HCMV disease, but not against wild-type HCMV infection 102. The Toledo genome contains mutations disrupting RL13, UL9, UL36, and UL128, and a 14-kb inversion of UL/b' sequences encoding UL130 to UL148 103. Thus, due to disruption of UL128, Toledo resembles Towne by not expressing the PC and consequently lacks epithelial or endothelial cell tropism.

The second-generation vaccine, based on early investigation, the chimeras Towne and Toledo of the magnitude and duration of the serologic responses were not notably more rapid or more robust 103. To date, the most extensively studied vaccine is a subunit vaccine based on the viral envelope glycoprotein B (gpUL55) and tegument phosphoprotein 65(PP65). gB is an essential glycoprotein that plays a crucial role in virus binding to the cell surface, therefore it has been proposed as a target for the development of recombinant vaccines 104. Furthermore, pp65 is regarded as a significant target of cytotoxic T cell (CTL) 105, 106, and the most likely vaccine target candidate to induce CTL-mediated protection against HCMV diseases 107. The subunit gB vaccine was developed in the late 1980s and is composed of a Chinese hamster ovary (CHO) cell-derived protein admixed with an oil-in-water emulsion 108; such the MF59-adjuvanted gB protein subunit vaccine (gB/MF59) is still an important research topic 109, 110. In the design of the gB/MF59 vaccine, gB was truncated at the transmembrane domain, and the furin protease site was deleted to facilitate purification from CHO cell supernatants. The vaccine was found to be safe and immunogenic in phase I studies 111 and can enhance the neutralizing potency of antibodies against HCMV gB and immune sera under complement enhancement 112. Acanarypox vector-expressing CMV phosphoprotein 65 is the first recombinant vaccine to elicit CMV-specific CTL responses in humans 113. Ad-gBCMVpoly is a novel chimeric vaccine based on a replication-deficient adenovirus that encodes a truncated form of CMV-encoded gB antigen and multiple CMV T-cell epitopes from eight different CMV antigens, restricted through multiple human leukocyte antigen (HLA) class I and class II alleles, as a single fusion protein 114. An enveloped virus-like particle (eVLP) vaccine expressing full-length or chimeric HCMV gB protein was generated by Variation Biotechnologies Incorporated (VBI) laboratories, where the extracellular domain (ECD) of gB is membrane-anchored using the transmembrane and cytoplasmic domains of the vesicular stomatitis virus G protein 115. eVLP was shown to induce a neutralizing antibody response 10-fold higher than its soluble recombinant protein counterpart 115. Therefore, a vaccine against the combination of gB, pp65, or US28 (which seems to be a more significant antigen of HCMV) may be more efficacious.

The third-generation vaccine ASP0113 (Astellas Pharma, Tokyo, Japan), a DNA vaccine containing two plasmids encoding CMV antigens (gB aimed at inducing the production of antibodies against CMV and PP65 inducing T-cell mediated responses), was found to be safe and well tolerated in Japanese recipients undergoing allogeneic hematopoietic stem-cell transplantation (HCT). VCL-CB01, a candidate CMV DNA vaccine that contains plasmids encoding CMV pp65 and gB to induce cellular and humoral immune responses, is formulated with poloxamer CRL1005 and benzalkonium chloride to enhance immune responses 116. The data regarding CMVPepVax, a novel HCMV peptide vaccine formulated with the TLR9 agonist adjuvant PF03512676, provide proof of concept that an HCMV vaccine, in the HCT setting, can increase the number of pp65-specific CD8 T cells, protect from HCMV reactivation, and reduce the use of antivirals 117. VCL-CT02, a trivalent HCMV DNA vaccine consisting of three plasmids, VCL-6368 (encoding pp65), VCL-6365 (encoding exon 2 and exon 4 of the IE1 gene), and VCL-6520 (encoding the extracellular domain of CMV Gb), appears to safely prime for a memory response to CMV antigens observed after administration of a live, attenuated CMV, and the strength of CMV antigen-specific immune response correlates with the priming effect of the DNA vaccine as measured by a cultured IFN-γ ELISPOT assay 118.

Some scholars propose that an mRNA-based gB vaccine may ultimately prove more efficacious than some second-generation vaccines and increase the durability and breadth of vaccine-elicited antibody responses that can prevent HCMV-associated disease 119. Nevertheless, the gB vaccine is not entirely reliant on the classic biological activity of neutralization 120. These potent vaccines are a composite of anti-gB and other major glycoprotein targets, including the trimer gH/gL/gO and the pentameric complex, which may explain the success of the gB HCMV vaccine. However, many molecules, including IE1, IE2, and pp65, have been used as targets for the development of CMV vaccines and the application of vaccine-based immunotherapy 121-123.

## Adoptive cell therapy

Adoptive cell therapy (ACT) involves the extraction of immune cells from the body, in vitro genetic modification and amplification, and transfusion back into the patient 124, 125, as a personalized immunotherapy 126. At present, there are four adoptive cell therapies that have made great progress in international research, including tumor-infiltrating lymphocytes (TILs), T-cell receptor (TCR)-engineered T cells, chimeric antigen receptor (CAR) T cells 127, and natural killer (NK) cell therapy 125. In 1992, adoptive CMV-specific T-cell therapy against viral infections achieved its first clinical application 128. Two different strategies have been developed to enrich, isolate, or purify virus-specific T cells 129. The one is in vitro stimulation and expansion of virus-specific T cells; autologous dendritic cells (DCs) pulsed with viral lysate were used as antigen-presenting cells (APCs) to stimulate CMV-specific T cells in vitro 130, then cells can either be used for in vitro expansion or isolation and direct infusion into the patient. By stimulation and amplification in vitro, a large number of virus-specific T cells can be obtained from a small amount of blood 129. The second is the direct selection of virus-specific T-cells. For the direct selection of virus-specific T cells, donor white blood cells are isolated ex vivo via peptide-HLA multimers, cytokine-capture method 131, 132, exposure to viral antigens, or methods based on the expression and upregulation of activation molecules on the surface of T cells 133. Virus-specific T cells are obtained in small amounts and infused into patients where they can expand efficiently and induce viral clearance as well as sustained protection.

ACT has become a therapeutic strategy for HCMV reactivation in patients undergoing allogeneic hematopoietic stem cell transplantation (HSCT) 134, 135 and solid organ transplant (SOT) 136. For instance, in patients with glioblastoma multiforme (GBM), CMV antigens were found in GBM tissues but not surrounding healthy brain cells 15, suggesting that HCMV plays a key role in glioblastoma and has implications for immunotherapeutic strategies 137. HCMV promotes murine glioblastoma growth via pericyte recruitment and angiogenesis. A study has shown direct killing of primary GBM cells by autologous HCMV-specific T cells 138, and some investigators developed a novel adoptive immunotherapy approach targeting CMV antigens for patients with recurrent GBM, and experimental results showed that autologous T-cell therapy was completely safe and associated with extended progression-free survival in 4 out of 10 patients 139. Furthermore, adoptive transfer of virus-specific T cells (VSTs) to achieve antiviral protection for patients treated with allogeneic HSCT resulted in response rates for HCMV of 94% 140. These results also demonstrate that CMV-specific T cells can effectively target glioblastoma cells for immune killing and support the theoretical basis for the development of CMV directed immunotherapy in patients 138. Bringing forward strategies of adoptive T-cells promoted the application of transplantation in the treatment of refractory viral infections after HSCT 129. In clinical application, a 21-year-old female patient with acute myeloid leukemia (AML), was treated with adoptive HCMV-specific T cells from her HLA-haploidentical sister, indicating that HCMV replication could be intermittently controlled by VSTs from an HCMV-positive donor 141. In recent years, there has been numerous clinical evidence for adoptive HCMV-specific T cell immunotherapy that has achieved satisfactory therapeutic effects 134, 135, 142-144. These studies strongly suggest that adoptive cellular immunotherapy is a safe and effective approach for treating cancer patients with severe HCMV infections in the future. Indeed, the application of immunotherapy not only provided a new method to anti-CMV strategies, but it is also worthy to be considered in other treatments of refractory viral infections, such as Epstein-Barr virus (EBV), adenovirus (AdV), and even multivirus infections 135, 143, 144.

## Oncolytic viruses

CMV is a unique oncolytic virus that is produced by genetic modification of some virulent viruses existing in nature. It uses the inactivation or defect of tumor suppressor genes in target cells to specifically recognize and infect tumor cells, replicate in large quantities, and eventually destroy tumor cells. It also stimulates an immune response that attracts more immune cells to continue killing the remaining cancer cells, and this is a growing field aimed to identify new therapies for treating cancer. Recently, there has been interest in developing vaccines based on CMV, which can induce large numbers of CD8+ T cells that are specific for an epitope that the virus encodes, better known as memory inflation 53, 145-148, thus it can be manipulated to express genes of interest for vaccination 146. CMV vector expressing the NKG2D ligand RAE-1γ, as a CD8+ T-cell-based anti-malignancy vaccine, could delay tumor growth and even provide complete protection against tumor attack during prevention and treatment 149.

For a lot of “cold tumors,” tumors with low immunogenicity, the response to traditional methods such as radiotherapy, chemotherapy, and immunotherapy is limited 150; Recent studies have demonstrated that the response is correlated with T cell infiltration and an “inflamed” tumor phenotype 151, 152. Thus, some scholars have applied CMV-based vaccine methods for converting “non-inflamed” to “inflamed” tumors combined with adoptive T cellar immunotherapy to treat this subset of patients. For instance, MCMVgp100KGP vaccine targeting melanoma gp100 antigen was generated in a laboratory utilizing recombinant murine CMV as a vaccine carrier, gp100 specific CD8+ T cells were activated by the vaccine to effectively protect mice against highly aggressive lung B16-F10 melanoma 153. Furthermore, MCMVgp100KGP vaccine combined with adoptive T cell therapy have also showed CMV-based vaccines are effective therapies against immunosuppressive solid tumors 154. The reason why recombinant CMV as a vaccine vector is that CMV-specific CD8+ effector/effector memory T cell populations are large^55^, highly dynamic 155, strong cytotoxic effect 156, and have the effect of a longer duration 155, 156. Therefore, using HCMV as an oncolytic virus to activate the immune system response is beneficial for both the anti-tumor and potentially antiviral response, and ultimately alleviates the disease and may cure the patient.

Whether the CMV can be used as an oncolytic virus is a question that needs to be considered and solved. We have to consider whether CMV has oncogenic properties, infects other normal cells, and produces severe viral infections that exacerbate the disease.

## Checkpoint blockade therapy and targeted antibodies

HCMV viremia in renal transplant recipients also appears to upregulate PD-1 expression on CD4 T cells 157. Studies have indicated that the use of immune checkpoint inhibitors (ICIs) can restore immune function and cause an immune response to CMV antigen when the infection is still latent 158. The pS-CIFT-aPD-1 is a vector for the expression of the anti-programmed cell death protein 1 (anti-PD-1) antibody gene under the control of a chimeric promoter composed of the CMV enhancer, the core IFN-γ promoter and human T-lymphotropic virus long terminal repeat sequence (TLTR), which co-transfected with the CAR construct into T cells showed increased production of anti-PD-1 antibodies, increased release of IFN-γ, greater T cell activation, and superior antitumor activity 159.

Targeted antibodies are antibodies designed to target antigens on cancer cells, primarily to disrupt the unrestricted growth and proliferation of infected cells. One approach uses antibody peptide epitope conjugates (APECs) to deliver suitable antigens to the tumor surface, which directs pre-existing CMV immunity against tumor cells and activates specifically CMV-reactive effector T cells, whereas a bispecific T-cell engager activates both effector and regulatory T cells 160. To eliminate the virus, it is more important to activate cellular immunity, whereas antibodies play crucial roles in preventing HCMV spreading in the blood.

## Conclusions and future directions

In general, it is obvious that the development of vaccines designed to target HCMV proteins as antigens and stimulate the body to produce effective antibodies and immunocytes has still a long way to go. In adoptive immunotherapy, HCMV is often used as a target antigen to activate anti-CMV-specific T lymphocytes, thereby killing tumor cells infected with CMV. In terms of immune checkpoints, HCMV infection upregulates the expression of genes encoding immune checkpoint genes as well as other immunocyte markers. Although antibody therapy is very limited for CMV because of its intracellular presence in a latent form, interrupting the FcR-associated immune escape pathway and targeting immune checkpoints are promising strategies. In oncolytic virus therapy, CMV is often used as a vector to express oncogenes and induce the anti-tumor ability of T lymphocytes. Of course, due to safety concerns, research in the area of oncolytic viruses is cautious, and the existing research is based on cell and animal models, instead of patients.

HCMV continues to be one of the most significant pathogens affecting the short-term and long-term outcomes of immunocompetent and immunocompromised patients. Firstly, HCMV asymptomatic latent infection and its severity once reactivated are the main obstacles of fundamental research and clinical therapeutics. Considering standardization and personalized immunotherapy, it is difficult to design a relatively perfect immunotherapy regimen to account for the particular complexity of commonly infected patients, different organ transplants, the heterogeneity of tumors (hot and cold tumors), and co-infection with other pathogens simultaneously. Secondly, identification of targets for HCMV immunotherapy, clinically meaningful inclusion criteria, and markers of immune responses, such as peripheral blood cell changes and inflammatory cytokines (IL-6, IFN-γ, TNF-α, IFN-α/β, etc.) is crucially important. Thirdly, the efficacy of single-target immunotherapy or single immunotherapy is limited. However, multi-target or multi-combination immunotherapy may cause unpredictable inflammatory cell infiltration, even cytokine storm, which are worthy of consideration. Finally, advances in immunotherapy technology coupled with the recent fundamental advances in the understanding of HCMV infection have created opportunities for the development of effective immunotherapy for HCMV infection. It is likely that combinatorial regimens with complementary mechanisms of action are required to achieve a broad and durable anti-HCMV benefit.

## Abbreviations

HCMV: human cytomegalovirus
HHV-5: human herpesvirus 5
CMV: cytomegalovirus
AIDS: Acquired Immune Deficiency Syndrome
GCV: ganciclovir
CDV: Cidofovir
FOS: Foscavir
dsDNA: double-stranded DNA
UL: unique sequence of long component
US: unique sequence of short component
TRL: terminal repeat of long component
IRL: inverted repeat of long component
IRS: inverted repeat of short component
TRS: terminal repeat of short component
IE: immediate-early gene
E: early gene
L: late gene
gB: Glycoprotein B
gH: glycoprotein H
gL: glycoprotein L
gM: glycoprotein M
gN: glycoprotein N
IL-1β: interleukin-1β
TC: trimeric gH/gL/gO complex
PDGFRα: platelet-derived growth factor receptor alpha
PC: pentameric gH/gL/UL128-131 complex
Nrp2: neuropilin-2
MTs: microtubules
MIEP: major immediate early promoter
EMT: epithelial mesenchymal transition
ROCK: Rho-associated coiled-coil kinase
LAT: latency-associated transcript
LUNA: latent undefined nuclear antigen
ERK: extracellular signal-regulated kinase
MAPK: mitogen activated protein kinase
TGF-β: transforming growth factor-β
BAFF-R: B cell-activating factor
NK cells: natural killer cells
TLR: toll-like receptors
STING: stimulator of interferon genes
cGAS: cyclic GMP/AMP synthase
TBK1: TANK-binding kinase 1
IRF3: interferon regulatory factory
PD-1: programmed cell death protein 1
PD-L1: programmed death ligand-1
TIM-3: T cell immunoglobulin domain and mucin domain-3
LAG-3: Lymphocyte-activation-gene-3
HLTF: Helicase-like transcription factor
TP53BP1: tumor protein p53-binding protein 1
ADCP: antibody-dependent cellular phagocytosis
FcRn: Fc receptor functions
PP65: phosphoprotein 65
CTL: cytotoxic T cell
CHO cell: Chinese hamster ovary
gB/MF59: MF59-adjuvanted gB protein subunit vaccine
HLA: human leukocyte antigen
eVLP: enveloped virus-like particle
HCT: hematopoietic stem-cell transplantation
ACT: adoptive cell therapy
TILs: tumor-infiltrating lymphocytes
TCR: T-cell receptor
CAR: chimeric antigen receptor
DC: dendritic cells
APCs: antigen-presenting cells
HSCT: hematopoietic stem cell transplantation
SOT: solid organ transplant
GBM: glioblastoma multiforme
VSTs: virus-specific T cells
EBV: Epstein-Barr virus
AdV: adenovirus
TLTR: T-lymphotropic virus long terminal repeat sequence
APECs: antibody peptide epitope conjugates
TAP: transporter associated with antigen processing
